# New Biological and Chemical Evidences of Two Lamiaceae Species (*Thymbra capitata* and *Thymus sipyleus* subsp. *rosulans*): In Vitro, In Silico and Ex Vivo Approaches

**DOI:** 10.3390/molecules27249029

**Published:** 2022-12-18

**Authors:** Eulogio J. Llorent-Martínez, Antonio Ruiz-Medina, Gokhan Zengin, Gunes Ak, Sharmeen Jugreet, Mohamad Fawzi Mahomoodally, Gizem Emre, Giustino Orlando, Maria Loreta Libero, Nilofar, Alessandra Acquaviva, Simonetta Cristina Di Simone, Luigi Menghini, Claudio Ferrante, Luigi Brunetti, Lucia Recinella, Sheila Leone, Mohamad Ali Shariati, Abdullahi Ibrahim Uba, Annalisa Chiavaroli

**Affiliations:** 1 Department of Physical and Analytical Chemistry, Faculty of Experimental Sciences, University of Jaén, Campus Las Lagunillas S/N, E-23071 Jaén, Spain; 2 Department of Biology, Science Faculty, Selcuk University, Konya 42300, Turkey; 3 Department of Health Sciences, Faculty of Medicine and Health Sciences, University of Mauritius, Réduit 80837, Mauritius; 4 Center for Transdisciplinary Research, Department of Pharmacology, Saveetha Institute of Medical and Technical Science, Saveetha Dental College, Chennai 600077, India; 5 Centre of Excellence for Pharmaceutical Sciences (Pharmacen), North-West, Private Bag X6001, Potchefstroom 2520, South Africa; 6 Department of Pharmaceutical Botany, Faculty of Pharmacy, Marmara University, Istanbul 34854, Turkey; 7 Botanic Garden “Giardino dei Semplici”, Department of Pharmacy, “Gabriele d’Annunzio” University, Via dei Vestini 31, 66100 Chieti, Italy; 8 Department of Scientific Research, K.G. Razumovsky Moscow State University of Technologies and Management (The First Cossack University), 73 Zemlyanoy Val, Moscow 109004, Russia; 9 Department of Scientific Research, Russian State Agrarian University—Moscow Timiryazev Agricultural Academy, 49 Timiryazevskaya Str., Moscow 127550, Russia; 10 Department of Molecular Biology and Genetics, Faculty of Engineering and Natural Sciences, Kadir Has University, Istanbul 34083, Turkey

**Keywords:** *Thymbra capitata*, *Thymus sipyleus* subsp. *rosulans*, phenolic, flavonoid, antioxidant, enzyme inhibitors, anti-inflammatory

## Abstract

In this study, the methanolic and infusion extracts of two species, *Thymbra capitata* and *Thymus sipyleus* subsp. *rosulans*, were tested for their chemical composition and biological abilities (antioxidant, enzyme inhibitory and anti-inflammatory effects). The extracts yielded total phenolic and flavonoid contents in the range of 83.43–127.52 mg GAE/g and 9.41–46.34 mg RE/g, respectively. HPLC analysis revealed rosmarinic acid to be a major component of the studied extracts (15.85–26.43%). The best ABTS radical scavenging ability was observed in the methanol extract of *T. capitata* with 379.11 mg TE/g, followed by in the methanol extract of *T. sipylus* (360.93 mg TE/g). In the CUPRAC assay, the highest reducing ability was also found in the methanol extract of *T. capitata* with 802.22 mg TE/g. The phosphomolybdenum ability ranged from 2.39 to 3.61 mmol TE/g. In terms of tyrosinase inhibitory effects, the tested methanol extracts (83.18–89.66 mg KAE/g) were higher than the tested water extracts (18.74–19.11 mg KAE/g). Regarding the BChE inhibitory effects, the methanol extracts were active on the enzyme while the water extracts showed no inhibitory effect on it. Overall, the methanolic extracts showed better enzyme inhibition compared to the infusion extracts. Molecular docking also showed the selected exhibited potential binding affinities with all enzymes, with a preference for cholinesterases. Additionally, the extracts were effective in attenuating the LPS-induced increase in COX-2 and IL-6 gene expression in isolated colon, thus indicating promising anti-inflammatory effects. The preliminary results of this study suggest that these species are good natural sources of antioxidants and also provide some scope as enzyme inhibitors, most likely due to their bioactive contents such as phenolic acids, and thus can be exploited for different applications related to health promotion and disease prevention.

## 1. Introduction

Species of the Lamiaceae family possess exceptional beneficial attributes and have diverse applications as functional foods, including pharmaceutical and cosmetic ingredients. It is well known that each species has distinct and complex combinations of bioactive constituents, with each component contributing to its overall bioactivity. Their value is due to their capacity to produce a range of secondary metabolites with strong antioxidant, anti-inflammatory, antimicrobial, antiviral, and anticancer activities, among others. Since ancient times, a variety of species of the Lamiaceae family have enjoyed a rich tradition of use as food preservatives, flavors and for curative purposes, because of both their therapeutic and preventive properties [1,2,3]. In this context, new studies on the members of the Lamiaceae family could provide innovative applications.

One famous plant species from this family is thyme, which has been appraised for its economic value [4]. The essential oils from various aromatic species of the thyme genus have been studied for their phytochemical composition and possible pharmacological applications [1,2,3,4,5,6].

Interestingly, *Thymbra* and *Thymus* species are common in the west Mediterranean area, which is believed to be the center of origin of the genus *Thymus*, and further extend westwards into the Iberian Peninsula and northwest Africa, to the Macaronesian area in the Atlantic Ocean [4]. Indeed, several works have been documented on the representatives of these genera, which include chemotaxonomy, antimicrobial and antioxidant activities of their volatile-containing extracts as well as essential oils [1,4]. In the literature, different yields for essential oils such as 1.78% for *Thymus atlanticus* [5], 0.06–2.8% for *Thymus algeriensis* [6] and 2.4–4.8% for *Thymbra capitata* [7] were obtained. Regarding extracts, the extraction yields varied depending on the solvent used. For example, in a recent study by Yassin et al. [8], the yields ranged from 0.45% (in n-hexane) to methanol (11.54%) for *Thymus vulgaris*. The extraction yields for hydroethanolic extracts of *Thymus citriodorus* and *Thymus vulgaris* were 14.05% and 24.34%, respectively [9].

In various parts of the world, these species have also been widely employed in traditional medicine since historical times. For instance, they have been used to treat digestive and respiratory system ailments. In ancient Egypt, they were used to make fragrant balms for embalming and other therapeutic purposes, while in Greece, thyme was utilized against asthma and to decongest the throat [10]. Thyme is also collectively used with other herbs to cure a range of illnesses, from sore throat and bronchitis to gastritis and skin illnesses. Thyme tea, drunk regularly, is even known to relieve arthritis [11]. The decoction and infusion of thyme’s aerial parts is used as a tonic, digestive, carminative, antispasmodic, and expectorant and for treating colds [12]. Regarding its pharmacological properties, scientific investigations have revealed that they possess an array of health benefits, including antimicrobial, antioxidant, anti-inflammatory, antiviral, antinociceptive and anti-cancer activities [10,13,14].

Similarly, *T. capitate,* which also extends over a wide range in the Mediterranean region, has been used to treat all types of diseases. It is used as a herbal tea, condiment and food additive and the plant oil is incorporated into soups, salads and pastries. In folk medicine, it is known to be used for colic, ulcers and hypertension [15]. It also has the characteristic of eliminating warts, being diuretic and actuating menstrual discharge. Besides, its leaves have antiseptic and purgative abilities. *Thymbra capitata* (L) is largely recognized for its antibacterial, antimycotic, antioxidant and spasmolytic potentials. It is broadly used in the pharmaceutical, cosmetics and food industries owing to its phenol and terpene contents [15,16,17].

Hence, considering the medicinal benefits of thyme, the main purpose of this study was to further investigate the chemical compositions and biological activities (antioxidant, enzyme inhibitory and anti-inflammatory) of two extracts (methanol and water) from two species, namely *T. capitata* and *T. sipyleus* subspecies *rosulans.*

## 2. Results and Discussion

### 2.1. Total Phenolic and Flavonoid Content

Phenolic compounds as well as flavonoids are well-known bioactive agents that have been extensively reviewed and have attracted considerable attention owing to their versatile benefits to human health and in curing and averting numerous illnesses [18].

Flavonoids and phenolic compounds are plant secondary metabolites that possess an aromatic ring having at least one hydroxyl group. They have been reported to be effective antioxidants, antibacterial, anticancer, cardioprotective, anti-inflammation, immune system promoting agents, skin protectors, and are therefore outstanding candidates for pharmaceutical and medical purposes [19,20]. Given their importance to plants and human health, it is considered useful to have an improved understanding of flavonoid contents and biological properties, which could be indicative of their potentials as healing agents, and also for predicting and establishing the quality of medicinal herbs [21]. Hence, the preliminary investigation of the presence of phenolics and flavonoids for the tested plants was determined by spectrophotometric assays.

Indeed, along with the extraction techniques used to recover antioxidant compounds from plants, the type of the solvent used is also important in determining the extraction yield of bioactive contents. In this regard, polar solvents are frequently used for recovering polyphenols from plant matrices [22]. Thus, in this study, the polar solvents water and methanol were used in extraction methods, notably infusion and maceration, respectively.

In this study, total phenolics and flavonoids contents were shown by all extracts (TPC: 83.43–127.52 mg GAE/g; TFC: 9.41–46.34 mg RE/g). However, the extracts of *T. capitata* were shown to be higher in TPC than *T. sipyleus* extracts. On the other hand, the *T. capitata* infusion extract yielded the least TFC compared to the other extracts. As previously reported, a variety of antioxidant compounds with different chemical characteristics and polarities may or may not be soluble in a particular solvent [22], therefore suggesting the lower TFC yield in *T. capitata* infusion extract compared to the other extracts. In addition, all the extracts were found to possess potent radical scavenging capacity, as revealed by the DPPH and ABTS assays (240.73–269.71 mg TE/g and 305.60–379.11 mg TE/g, respectively) (Table 1).

### 2.2. Chemical Characterization

#### 2.2.1. Phenolic Acids

Phenolic acids, a subclass of plant phenolics, possess a phenolic moiety and a resonance-stabilized structure, which makes the H-atom donation responsible for their antioxidant properties through the radical scavenging mechanism. Other modes, such as radical quenching by electron donation and singlet oxygen quenching, are also recognized for the antioxidant activity of phenolic acids. Additionally, phenolic acids are ubiquitous and known for their protective health effects, such as anticancer, anti-inflammatory, antimicrobial and anti-mutagenic properties [23]. Furthermore, many of the *Lamiaceae* species, including thymus species, are reported to be rich in bioactive compounds, particularly phenolic acids [24], making their characterization important in the present study. Following is a description of the identification of phenolic acids in the analyzed extracts (Table 2).

Compound **5** suffered the neutral loss of 162 Da (hexoside) to yield the MS^2^ base peak at *m*/*z* 153. The fragmentation of the ion at *m*/*z* 153 was consistent with dihydroxybenzoic acid (an analytical standard of protocatechuic acid, dihydroxybenzoic acid, was used to compare the fragmentation pattern). Hence, it was characterized as dihydroxybenzoic acid-*O*-hexoside.

Compounds **7** and **8** presented the fragmentation pattern typical of caffeoylquinic acids. Specifically, compound **8** was identified as 4-*O*-caffeoylquinic acid by comparison with an analytical standard. The presence of caffeoylquinic acids in *Thymus* species has been previously reported [25], although chlorogenic acid was mentioned as the found isomer.

Compounds **9**, **16** and **19** were identified as feruloylquinic acids. The corresponding isomers were assigned according to the hierarchical scheme proposed in [26]. The presence of feruloylquinic acid has been reported in *T. zygis* [27]. Compounds **12**, **17** and **47** were tentatively characterized as ferulic acid derivatives due to the presence of ferulic acid at *m*/*z* 193 (fragment ions at *m*/*z* 149 and 134).

#### 2.2.2. Flavonoids

Several apigenin *C*-glycosides were observed in the analyzed extracts. Compound **15** was identified as vicenin-2 (apigenin-6,8-di-*C*-glucoside) by comparison with an analytical standard. Compounds 20 and 24 were 6,8-di-C-asymmetricglycosyl apigenins. The differentiation of the isomers was performed by the different abundance of the fragment ion at *m*/*z* 545 (more abundant in 6-C-pentoside-8-C-hexoside) and the retention time [28]. Compound **21** presented fragment ions at *m*/*z* 431, 341 and 311, typical of vitexin (8-*C*-glucosyl apigenin); with an additional 162 Da, it was tentatively characterized as vitexin-hexoside. Similarly, compound **22** was characterized as vitexin-rutinoside. Compound **28** was vitexin (8-*C*-glucosyl apigenin) [21]. Compound **35** suffered the neutral loss of 162 Da to yield apigenin at *m*/*z* 269 (main fragment at *m*/*z* 225), so it was identified as apigenin-*O*-hexoside. The aglycone apigenin was compound **58**.

Compounds **18** and **25** suffered neutral losses of 162 (hexoside) and 308 Da (rutinoside) to yield the aglycone eriodictyol at *m*/*z* 287 (main fragment at *m*/*z* 151). The aglycone eriodictyol was compound **48**.

Compounds **27** and **32** displayed neutral losses of 162 and 176 Da to yield the aglycone luteolin at *m*/*z* 285 (fragment ions at *m*/*z* 241 and 243), so they were identified as luteolin-*O*-hexoside and luteolin-*+6*-glucuronide, respectively. Compound **51** was luteolin.

Compound **29** was tentatively characterized as taxifolin based on bibliographic information [29].

Compound **31** suffered the neutral loss of 162 Da to yield quercetin at *m*/*z* 301 (fragment ions at *m*/*z* 179 and 151), so it was identified as quercetin-*O*-hexoside. Compound **50** was quercetin (identified by comparison with an analytical standard).

Compound **34** was identified as naringenin-*O*-hexoside (loss of 162 Da), whereas **57** was the aglycone naringenin: deprotonated naringenin at *m*/*z* 271 and base peak at *m*/*z* 151. Compound **37**, which displayed two consecutive losses of 176 Da (glucuronide) was characterized as naringenin-di-*O*-glucuronide.

Compound **40** was identified as hesperidin by comparison with an analytical standard. Its presence has been previously reported in *T. vulgaris* [25].

#### 2.2.3. Rosmarinic and Derivatives (Salvianolic Acids)

Compound **42** was characterized as rosmarinic acid, whereas **33** was rosmarinic acid-*O*-hexoside. Both compounds have been previously reported in different *Thymus* species [25,30,31]. Several salvianolic acids and derivatives were detected, observing the fragment ion at *m*/*z* 359 (rosmarinic acid) in all of them.

Compound **43**, with a deprotonated molecular ion at *m*/*z* 555, displayed fragment ions at *m*/*z* 493, 359, 197, 179 and 161, characteristic of salvianolic acid K, previously reported in *T. algeriensis* [31] and *T. mastichina* [32]. Here, it was only found in *T. sipylus*, but not in *T. capitata*.

Compounds **44**, **46** and **52**, with a similar fragmentation pattern, were characterized as salvianolic acid B/E isomers. Their presence has been reported in *T. capitatus* [30], *T. algeriensis* [31] and *T. mastichina* [32]

Compound **45** was identified as salvianolic acid I (lithospermic acid A), previously described in *T. mastichina* [32] and *T. algeriensis* [31].

Compound **53** was tentatively characterized as salvianolic acid A due to the fragmentation pattern [33]. It has been reported in *T. mastichina* [32], although not with the same fragment ions.

Compound **55** was tentatively characterized as monomethyl lithospermate, reported in *T. alsarensis* [34] and also displayed the presence of rosmarinic acid at *m*/*z* 359.

#### 2.2.4. Other Compounds

Compound **1** was characterized as a disaccharide (HCl adduct) due to the neutral loss of 162 Da (341→179) and the characteristic fragments of hexoside moieties from the fragment ion at *m*/*z* 179 [35].

Compound **2** was tentatively characterized as a quinic acid derivative (191/173 fragmentation), whereas compounds **3** and **4** were identified as isocitric and citric acid by comparison with a citric acid analytical standard.

Compound **6** was tentatively characterized as danshensu (dimer) [36]. This compound has been previously reported in *T. zygis* subsp. *gracilis* [27].

Compound **11** presented the same fragmentation pattern as medioresinol [37].

Compound **36** suffered the neutral loss of 162 Da to yield syringaresinol at *m*/*z* 579 [38].

Compounds **56** and **59** were characterized as the oxylipins oxo-dihydroxy-octadecenoic acid and trihydroxy-octadecenoic acid based on bibliographic information [39].

### 2.3. Relative Peak Areas and Heat Map

To verify which compounds were the most abundant in the analyzed extracts, the peak areas of each compound were obtained in MS mode using the precursor ion [M-H]^−^ (extracted ion chromatograms). The relative percentage of each compound was calculated by area normalization and is shown in Table 3, in which the heat map highlights the most abundant compounds.

In *T. sipyleus*, the most abundant compound was rosmarinic acid (compound **42**), which accounted for approximately 16% (infusion) and 26% (methanolic) of all compounds. It was followed by salvianolic acid K (4.6–10%) and salvianolic acid I (13%, but only found in the MeOH extract). Many other compounds had a similar contribution to the extract, such as luteolin-*O*-glucuronide (6–11%), medioresinol (5–7%), vicenin-2 (4–12%) and 4-feruloylquinic acid (4%). Thus, the main bioactivity of the extracts would be due to rosmarinic acid and salvianolic acids, but several phenolic acids and flavonoids would also contribute to the overall bioactivity.

Regarding *T. capitata*, rosmarinic acid was still abundant (21–23%). However, concerning salvianolic acids, B and E isomers were relatively abundant (compounds **44** and **46**; 5–10%), whereas salvianolic acids K and I were not detected. Vicenin-2 had a similar proportion to that in *T. sipyleus* (11–14%). However, the main difference was observed in compound **10**, which presented a high percentage and was not detected in *T. sipyelus*. The presence of a compound with deprotonated molecular ion at *m*/*z* 305 (compound **10**) has been previously reported in *T. fontanesii* [40] as gallocatechin. However, the fragmentation pattern observed here differs from that previously described as gallocatechin, so we could not identify it with confidence.

High-performance liquid chromatography (HPLC) analysis revealed that rosmarinic acid was the major component of the studied extracts (15.85–26.43%) (Table 3). Rosmarinic acid, a caffeic acid ester, is a naturally occurring phenolic compound found in a variety of plants that belong to the *Lamiaceae* family [24]. It is known to exhibit a range of pharmacological attributes, e.g., antioxidant, anti-inflammation, antiviral, antidiabetic, antitumor, including neuroprotection and hepatoprotection effects, as demonstrated in several in vivo and in vitro studies [41].

Vicenin-2 was also among the major components of the *T. capitata* extracts (10.98 and 13.98%) and infusion extract of *T. sipyleus* (12.23%), whereas salvianolic acid I was present among the major component in the methanolic extract of *T. sipyleus* (12.66%) (Table 3). Remarkably, a growing body of evidence suggests that these *Thymus* species are rich sources of bioactive compounds, including phenolic compounds such as rosmarinic and salvianolic acids and luteolin glycosides, making them attractive candidates for a variety of industrial applications [42].

In a recent review by Elbouny et al. [43], a *Thymus* species was found to be rich in phenolic compounds, both in its volatile oils as well as in its non-volatile extracts, including phenolic acids such as rosmarinic, salvianolic, caffeic and ferulic acids, and flavonoids luteolin, gallocatechin, isorhamnetin and quercetin, among others.

### 2.4. Chromatographic Quantification of the Main Phytochemicals

The quantification of the main compounds was performed by HPLC-DAD, using 320 nm for phenolic acids and 350 nm for flavonoids. Repeatability (*n* = 9) and intermediate precision (*n* = 9, 3 consecutive days) were lower than 4 and 9%, respectively. The quantification was performed using analytical standards of the corresponding family in each case and the results are given in Table 4. It can be observed that the TIPC was in the following order: *T. sipylus* (MeOH) > *T. sipylus* (Inf) > *T. capitata* (MeOH) > *T. capitata* (Inf). These results agree with Table 1 (the sum of TPC and TFC follows the same order). In addition, the main quantified compounds were rosmarinic acid, salvianolic acids, vicenin-2 and luteolin-O-glucuronide, in line with the findings in the semiquantification (Table 3).

### 2.5. Antioxidant Properties

Maintaining the balance between free radicals and antioxidants is indeed an important condition for remaining healthy. Therefore, controlling oxidative stress processes is crucial for curing many diseases, such as atherosclerosis, diabetes, cancer, inflammation, liver and cardiovascular diseases, cataracts, nephrotoxicity and age-related neurodegenerative developments [44].

Here, in addition to radical scavenging abilities, all extracts also demonstrated antioxidant potentials as reducing agents (CUPRAC: 622.65–802.22 mg TE/g; FRAP: 249.33–285.42 mg TE/g) and metal chelators (14.97–36.72 mg EDTAE/g) (Table 5). In particular, the *T. capitata* methanolic extract showed very high reducing activity in CUPRAC assay. Interestingly, the infusion extracts were found to be better metal chelators compared to the methanolic extracts. In addition, the total antioxidant capacity of the extracts was determined by the phosphomolybdenum assay, ranging from 2.39–3.61 mmol TE/g (Table 5).

Findings from various studies have highlighted that *Thymus* species are powerful natural antioxidants. For instance, in [45], six *Thymus* species were tested using six assays. All were reported to possess DPPH and nitric oxide scavenging activities (IC_50_: 3–6 μg/mL and 70–177 μg/mL, respectively), strong reducing properties, ferrous ion chelating activity and lipid peroxidation inhibition capacity (IC_50_: 11–15 μg/mL, 126–389 μg/mL, 34–80 μg/mL, respectively), including high total antioxidant capacities (238–294 mg AAE/g).

Furthermore, it has been reported that the antioxidant activity of *Thymus* species is closely linked with their phenolic abundance and/or specific phenolic contents [43,46]. In fact, the antioxidant properties of phenolic-rich extracts from a wide range of *Thymus* species have been documented in a recent review [42]. In addition, the decoction and infusion extracts of *T. sipyleus* Boiss. subsp. *rosulans* have been reported to contain the highest amount of phenolic content and showed the most potent activity against DPPH radical [47]. This is in agreement with the present findings given that relatively high TPC was produced in all extracts, which also showed potent antioxidant activities in the different assays conducted.

### 2.6. Enzyme Inhibition Properties

The inhibition of acetylcholinesterase (AChE) and butyrylcholinesterase (BChE) enzymes, which degrade acetylcholine, is considered a promising strategy for treating Alzheimer’s disease. A potent source of AChE and BChE inhibitors can be derived from an abundance of plants. In fact, natural products continue to provide valuable drugs and templates for the development of other compounds [48].

In the current study, all extracts were found to inhibit AChE (0.35–3.86 mg GALAE/g), although the methanolic extracts were more potent AChE inhibitors compared to the infusion extracts, whereas only the methanolic extracts inhibited BChE (*T. capitata*: 4.36 mg GALAE/g; *T. sipylus*: 3.79 mg GALAE/g) (Table 6).

The anti-AChE activity of the ethanolic extracts of six *Thymus* species was also examined by other authors [45], where all tested extracts showed AChE inhibitory activity in a dose-dependent way with extract concentrations of 0.25, 0.5 and 1 mg/mL showing inhibition values of 10–28%, 23–39% and 64–86%, respectively.

Even though melanin has principally a photoprotective role in human skin, the accumulation of an abnormal quantity of melanin in several parts of the skin can lead to the formation of more pigmented spots, causing an esthetic problem. On the other hand, enzymatic browning of fruits and fungi is normally undesirable. Post-harvest browning is a common phenomenon in crops and mushrooms, which decreases their market value. Hyperpigmentation of human skin and enzymatic browning of fruits are undesirable. These phenomena have prompted researchers to seek new potent tyrosinase inhibitors to combat food browning and skin depigmentation. Even though both natural and synthetic tyrosinase inhibitors have been found [48], there is still a high demand for more effective tyrosinase inhibitors, especially those made from natural sources. In the present study, all extracts were found to cause tyrosinase inhibition (18.74–89.66 mg KAE/g) (Table 6). However, methanolic extracts showed more potent tyrosinase inhibition compared to the infusion extracts.

The strategy of reducing carbohydrate digestibility by regulating the activity of two hydrolyzing enzymes, α-amylase and α-glucosidase, to control postprandial hyperglycemia is considered a viable prophylactic treatment for type 2 diabetes mellitus. Thus, the consumption of foods rich in hydrolyzing enzyme inhibitors is recommended for dietary therapy of diabetes [49]. Plants are indeed rich sources of these enzyme inhibitors, as shown in various reports [50,51,52], and can be implemented in dietary therapy as well as used for the development of phytomedicines for diabetes management.

In the present study, dual inhibition was demonstrated by all extracts against the carbohydrate digesting enzymes. All extracts inhibited amylase (0.11–0.84 mmol ACAE/g) and glucosidase (1.45–1.78 mmol ACE/g) (Table 6). The methanolic extracts showed relatively higher amylase and glucosidase inhibition compared to the infusion extracts.

Other *Thymus* species also showed antidiabetic potential through inhibition of amylase and glucosidase. These were *T. quinquecostatus* Celak, *T. schimperi* R., *T. vulgaris* L. and *T. persicus* [50,51,52].

### 2.7. Molecular Docking

**Figure 1:**
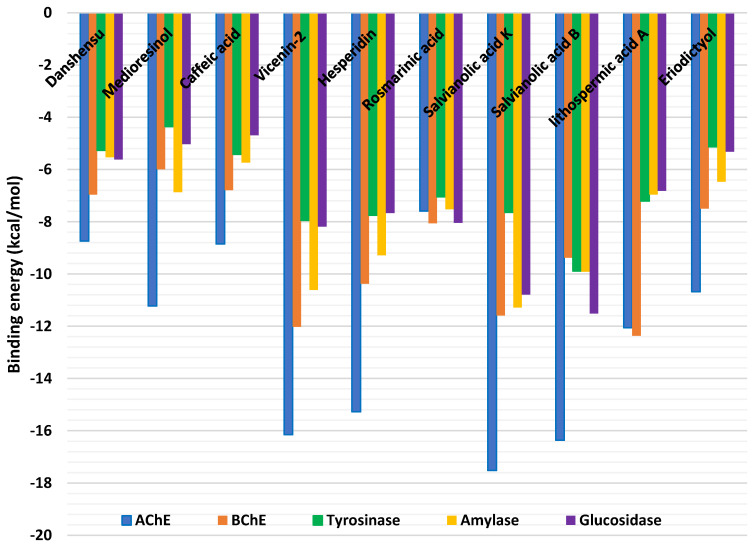
Binding energy (docking) scores of the phytochemicals from *Thymbra capitata* and *Thymus sipyleus* subsp. *rosulans* extracts.

**Figure 2:**
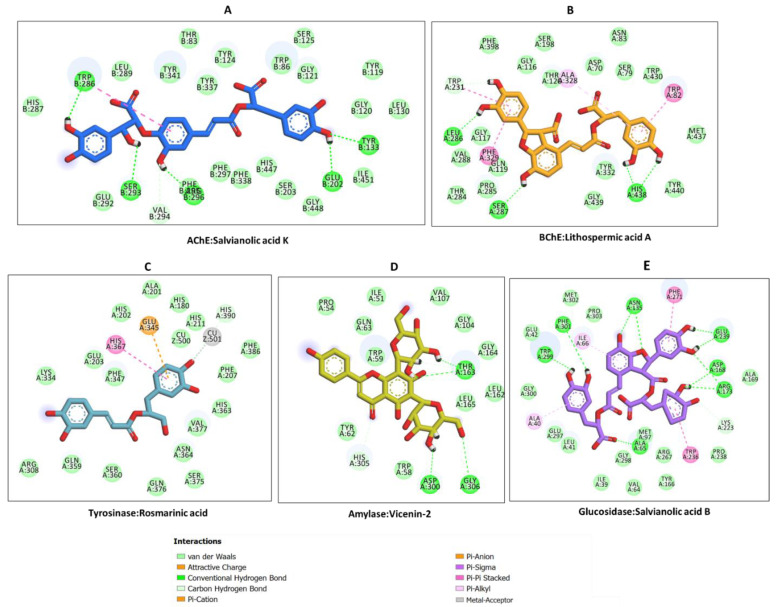
Protein–ligand interaction: (**A**) AChE and salvianolic acid K, (**B**) BChE and lithospermic acid A, (**C**) tyrosinase and rosmarinic acid, (**D**) amylase and vicenin-2, and (**E**) glucosidase and salvianolic acid B. The bioactive compounds were extracted from *Thymbra capitata* and *Thymus sipyleus* subsp. *rosulans*.

The docking (binding energy) score of each ligand against each target enzyme is shown in Figure 1. All compounds studied showed potential binding affinities for all five enzymes, with a preference, in particular, for AChE and BChE. Thus, the detailed protein–ligand interactions were visualized for some selected protein–ligand complexes. Salvianolic acid K is strongly bound to AChE via multiple H-bonds and van der Waals interactions all over the active site (Figure 2A). Salvianolic acid K is also bound to the rest of the enzymes with different levels of strength. Lithospermic acid A, a structurally related molecule to salvianolic acid K, bound BChE in an opposite orientation via a couple of π-π stacked, and a hydrophobic interaction, in addition to multiple H-bonds and van der Waals interactions that reinforced the binding (Figure 2B).

Rosmarinic acid occupied the tyrosinase catalytic channel with an interesting binding mode. Rosmarinic acid formed a metal acceptor interaction with the active site copper metal ion, a π-anion, a π-π stacked deep inside the tunnel and several van der Waals interactions throughout the active site of the enzyme (Figure 2C). On the other hand, the major interactions between amylase and vicenin-2 were H-bonds, formed deep in the pocket, with a couple of van der Waals interactions strengthening the binding (Figure 2D). Finally, salvianolic acid B was completely buried in the glucosidase catalytic cavity, and the key interactions formed comprised multiple H-bonds, a few π-π stacked and hydrophobic interactions and multiple van der Waals interactions along the entire length of the tunnel (Figure 2E). Together, these interactions are likely to allow these selected bioactive compounds extracted from *Thymbra capitata* and *Thymus sipyleus* subsp. *rosulans* to inhibit the biological activity of the target enzymes.

### 2.8. Ex Vivo Studies

**Figure 3:**
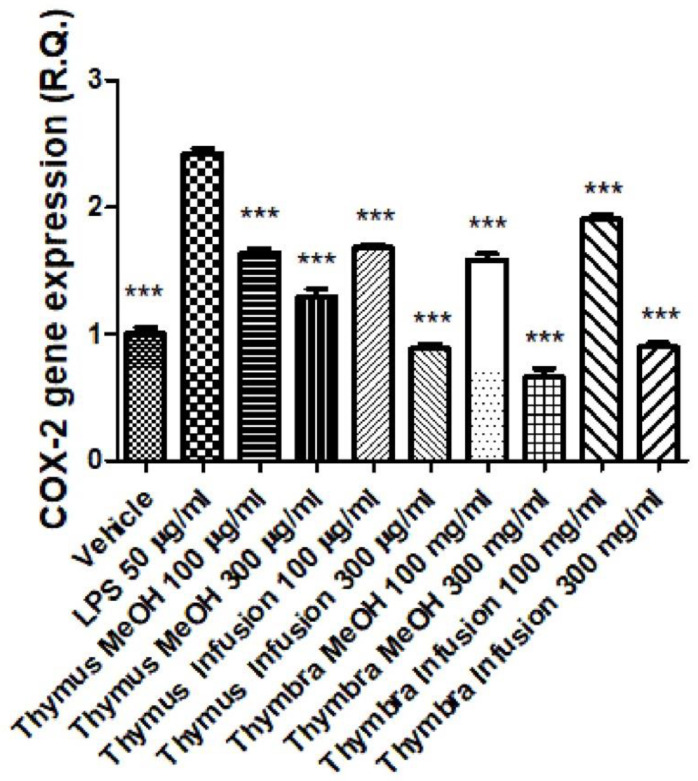
Inhibitory effects of *Thymus sipyleus* and *Thymbra capitata* extracts (100–300 µg/mL) on LPS-induced COX-2 gene expression, in isolated colon (ANOVA, *p* < 0.0001; *** *p* < 0.001 vs. LPS).

**Figure 4:**
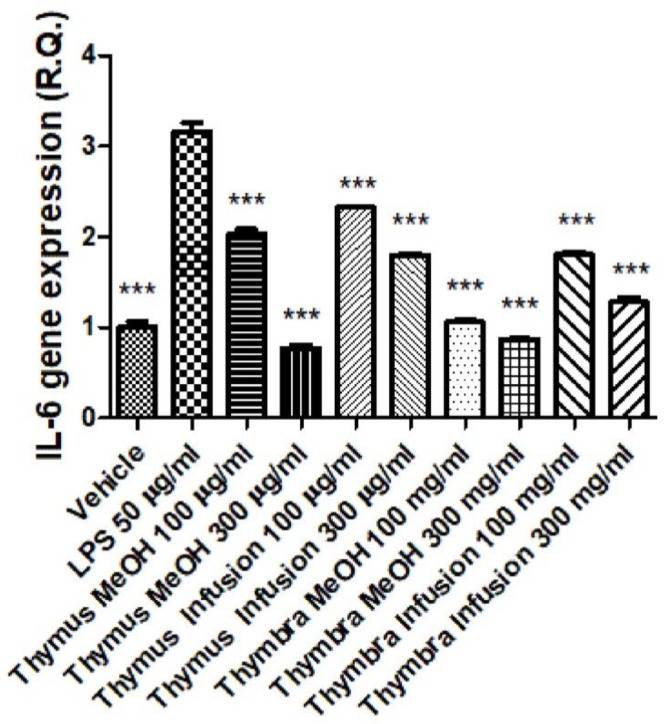
Inhibitory effects of *Thymus sipyleus* and *Thymbra capitata* extracts (100–300 µg/mL) on LPS-induced IL-6 gene expression, in isolated colon (ANOVA, *p* < 0.0001; *** *p* < 0.001 vs. LPS).

The extracts were also tested in an experimental model of inflammation consisting of isolated colon specimens exposed to LPS [53]. In this model, scalar concentrations of the extracts (100–300 µg/mL) were effective in attenuating the LPS-induced upregulation of both COX-2 and IL-6 gene expression (Figure 3 and Figure 4). These findings are consistent with the aforementioned intrinsic properties of the extracts, capable of functioning as both scavenging/reducing and enzyme inhibition agents. This is partly in agreement with the content of phenolic compounds [54,55]. The high content of rosmarinic acid, which has been shown to be an anti-inflammatory agent, is particularly evident; indeed, rosmarinic acid showed protective effects in in vivo models of ulcerative colitis induced by DSS, where the phytocompound was able to reduce the gene expression of COX-2 and IL-6 [56], possibly through the inhibition of NFkB activity.

Furthermore, it is rational to consider that other phenolic compounds present in the extracts, including vicenin-2, might play a key role in mediating the anti-inflammatory observed effects in mouse colon [57].

Collectively, these data indicate protective effects induced by *T. capitata* and *T. sipyleus* against colonic inflammation and partly corroborate the traditional use of these two Lamiaceae species as remedies for treating digestive disorders.

## 3. Materials and Methods

### 3.1. Plant Materials and Extraction

The aerial parts of the plants were collected from different regions of Turkey (*T. capitata*: Bozcaada, Canakkale, Turkey and *T. sipyleus* subsp. *rosulans*: Karagobek village, Erzurum, Turkey) during the summer season (at the flowering stage) of 2020. The plant was identified by one botanist co-author (Dr. Gizem Emre, Marmara University). Voucher specimens were deposited at the herbarium in the Marmara and Selcuk Universities.

In the preparation of plant extracts, we used two solvents (methanol and water) to extract compounds of different polarities. The maceration technique was selected for methanol extracts and for this purpose, plant materials (10 g) were stirred with the 200 mL of methanol for 24 h at room temperature. After that, the mixtures were filtered through Whatman filter paper and the solvents were removed using a rotary evaporator. Regarding water extracts, the extracts were prepared as a traditional infusion and the plant materials (10 g) were kept in boiled water (200 mL) for 15 min. Then, the mixture was filtered and lyophilized for 48 h. All extracts were stored at 4 °C until analysis.

### 3.2. Profile of Bioactive Compounds

The Folin–Ciocalteu and AlCl_3_ assays, respectively, were utilized to determine the total phenolic and flavonoid contents [58]. For the Folin–Ciocalteu assay, the sample solution (0.25 mL) was mixed with diluted Folin–Ciocalteu reagent (1 mL, ratio of 1:9) and shaken vigorously. After 3 min, Na_2_CO_3_ solution (1%, 0.75 mL) was added and the sample absorbance was read at 760 nm after 2 h incubation at room temperature. To determine the total flavonoid content, the sample solution (1 mL) was briefly mixed with the same volume of aluminum trichloride (2%) in methanol. Similarly, a blank was prepared by adding sample solution (1 mL) to methanol (1 mL) without AlCl_3_. The sample and blank absorbances were read at 415 nm after a 10 min incubation at room temperature. The absorbance of the blank was subtracted from that of the sample. For the respective assays, results were expressed as gallic acid equivalents (mg GAEs/g extract) and rutin equivalents (mg REs/g extract).

### 3.3. Instrumentation

Chromatographic analyses were performed using an Agilent Series 1100 HPLC system equipped with a G1315B diode array detector (Agilent Technologies) and an ion trap mass spectrometer (Esquire 6000, Bruker Daltonics) with an electrospray interface. Separation was performed in a Luna Omega Polar C_18_ analytical column (150 × 3.0 mm; 5 µm particle size) with a Polar C_18_ Security Guard cartridge (4 × 3.0 mm), both purchased from Phenomenex. Detailed chromatographic conditions are available in [59].

### 3.4. HPLC-ESI-MS^n^ Analysis

The characterization of the phytochemicals was carried out by HPLC-ESI-MS^n^ using the negative ion mode. Identification was performed using analytical standards as well as bibliographic information. Compounds were numbered according to their elution order, keeping the same numbering in all extracts. A brief explanation of the characterization of the compounds not identified by analytical standards follows.

### 3.5. Determination of Antioxidant and Enzyme Inhibitory Effects

The antioxidant and enzyme inhibitory activity of comfrey root extracts was determined according to previously described methods [60,61]. DPPH and ABTS radical scavenging activity, cupric ion reducing antioxidant capacity (CUPRAC) and ferric ion reducing antioxidant power (FRAP) were expressed as mg Trolox equivalents (TE)/g extract. The metal chelating ability (MCA) was reported as mg EDTA equivalents (EDTAE)/g extract, whereas the total antioxidant activity (phosphomolybdenum assay, PBD) was expressed as mmol TE/g extract. AChE and BChE inhibitory activities were given in mg galanthamine equivalents (GALAE)/g extract; tyrosinase inhibitory activity was expressed in mg kojic acid equivalents (KAE)/g extract; amylase and glucosidase inhibitory activities were presented in mmol acarbose equivalents (ACAE)/g extract.

### 3.6. Molecular Modeling

The crystal structures of target enzymes were downloaded from the protein data bank (PDB) (https://www.rcsb.org/ accessed on 1 June 2022): human AChE (6O52) [62], BChE (6EQP) [63] and human pancreatic alpha-amylase (1B2Y) [64]. Since the crystal structures of human tyrosinase and glucosidase have not yet been elucidated, those of Priestia megaterium tyrosinase (6QXD) [65] and Mus musculus alpha-glucosidase (7KBJ) [66] were used as templates to build their human models using UniProt sequences P14679 and P0DUB6, respectively.

The details of the model construction has been described elsewhere [67]. The prepared protein structures were taken from previous work [68]. The 3D structures of selected ligands were downloaded from the PubChem database (https://pubchem.ncbi.nlm.nih.gov/ accessed on 1 June 2022) and their geometry was optimized using Frog2 [69]. The respective cocrystal ligand of each complex was used to define the docking grid box dimension and binding coordinates using AutoDockTools 1.5.6, and docking was performed using AutoDock 4.2.6 (https://autodock.scripts.edu, accessed on 1 June 2022) [70]. The details of the docking, including the Lamarckian genetic algorithm employed and the number of runs, have been described previously [71,72,73,74]. The docking score of each ligand was calculated, and the protein–ligand interactions were visualized using Biovia Discovery Studio Visualizer (Dassault Systèmes Biovia Software Inc, 2012).

### 3.7. Ex Vivo Studies

Adult C57/BL6 male mice (3-month-old, weight 20–25 g) were housed in Plexiglas cages (2–4 animals per cage; 55 cm × 33 cm × 19 cm) and maintained under standard laboratory conditions (21 ± 2 °C; 55 ± 5% humidity) on a 14/10 h light/dark cycle, with ad libitum access to water and food.

Isolated colon specimens were collected from euthanized mice (Project no. F4738.N.5QP) and maintained in a humidified incubator with 5% CO_2_ at 37 °C for 4 h (incubation period), in RPMI buffer with added bacterial LPS (10 µg/mL), as previously described [53]. During the incubation period, the tissues were subjected to scalar concentrations of the extracts (100–300 μg/mL).

### 3.8. RNA Extraction, Reverse Transcription and Real-Time Reverse Transcription Polymerase Chain Reaction (RT-PCR)

Total RNA was extracted from colon specimens using TRI reagent (Sigma-Aldrich, St. Louis, MO, USA), according to the manufacturer’s protocol, and reverse transcribed using a High-Capacity cDNA Reverse Transcription Kit (ThermoFischer Scientific, Waltman, MA, USA). Gene expression was determined by real-time quantitative PCR using TaqMan probe-based chemistry. PCR primers and TaqMan probes were purchased from Thermo Fisher Scientific Inc. The Assays-on-Demand Gene Expression Products used for gene expression evaluations in the mouse colon specimens were: Mm00478374_m1 for COX-2 gene, Mm00607939_s1 for β-actin gene. β-actin was used as the housekeeping gene. The elaboration of data was conducted with the Sequence Detection System (SDS) software version 2.3 (ThermoFischer Scientific). Relative quantification of gene expression was performed by the comparative 2^−∆∆Ct^ method [75].

### 3.9. Statistical Analysis

In the antioxidant and enzyme inhibitory assays, the values are expressed as mean ± SD of three parallel experiments. To determine the differences between tested extracts in terms of antioxidant and enzyme inhibitory capacities, one-way ANOVA with Tukey test was performed. The statistical analysis was performed using XlStat 16.0 software.

In ex vivo studies, the software GraphPad Prism version 6.0 (Graphpad Software Inc., San Diego, CA, USA) was used to perform data analysis. Means ± SEM were determined for each experimental group and analyzed by one-way analysis of variance (ANOVA), followed by Newman–Keuls multiple comparison post hoc test. The limit of statistically significant differences between mean values was set at *p* value < 0.05. The number of animals randomized for each experimental group was calculated on the basis of the resource equation N = (E + T)/T (10 ≤ E ≤ 20) [76].

## 4. Conclusions

In this study, the methanolic and infusion extracts of *Thymbra capitata* and *Thymus sipyleus* subsp. *rosulans* were examined for their chemical composition and biological properties using in vitro, ex vivo and in silico studies. Spectrophotometric assays showed the extracts had higher total phenolic and flavonoid contents and high-performance liquid chromatography analysis revealed that rosmarinic acid was a predominant compound, which is in fact the characteristic of various *Thyme* species.

This compound could be the main one responsible for the anti-inflammatory effects of the tested extracts against LPS-induced toxicity in the mouse colon. While all extracts showed potent antioxidant capacity, most probably related to their relatively high TPC, their enzyme inhibition potency varied. For instance, the methanolic extracts were found to be better enzyme inhibitors compared to infusion extracts. This could be due to the absence of active components in the infusion extracts that are favorable for enzyme inhibition. The findings suggest that both species are good natural sources of antioxidants and can be used as enzymes inhibitors. Moreover, the herein presented chemical characterization and biological profile of these plants could help stimulate advanced research on their utility.

## Figures and Tables

**Table 1:** Total bioactive compounds and radical scavenging ability of the tested extracts.

Extracts	Total Phenolic Content (mg GAE/g)	Total Flavonoid Content (mg RE/g)	DPPH (mg TE/g)	ABTS (mg TE/g)
*T. capitata*-Methanol	127.52 ± 4.32 ^a^	44.08 ± 0.54 ^b^	269.71 ± 0.63 ^a^	379.11 ± 6.07 ^a^
*T. capitata*-Infusion	94.57 ± 0.83 ^b^	9.41 ± 1.58 ^d^	259.63 ± 0.62 ^b^	305.60 ± 7.56 ^d^
*T. sipylus*-Methanol	83.43 ± 0.57 ^d^	46.34 ± 0.27 ^a^	240.73 ± 3.49 ^c^	360.93 ± 1.61 ^b^
*T. sipylus*-Infusion	88.88 ± 0.65 ^c^	34.11 ± 2.50 ^c^	256.66 ± 5.12 ^b^	345.10 ± 4.33 ^c^

Values are reported as mean ± S.D of three parallel measurements. GAE: gallic acid equivalent; RE: rutin equivalent; TE: trolox equivalent. Different letters indicate significant differences in the tested extracts (*p* < 0.05).

**Table 2:** Characterization of the compounds found in the analyzed extracts of *Thymus sipylus* and *Thymbra capitata*.

					*T. sipylus*		*T. capitata*	
S. No.	t*_R_* (min)	[M-H]^−^ *m*/*z*	*m*/*z* (% Base Peak)	Assigned Identification	MeOH	Inf	MeOH	Inf
1	1.8	377	MS^2^ [377]: 341 (100) MS^3^ [377→341]: 179 (66), 161 (100), 143 (23)	Disaccharide (HCl adduct)	✓		✓	
2	1.8	533	MS^2^ [533]: 191 (100) MS^3^ [533→191]: 191 (100), 173 (28), 127 (14), 109 (12)	Quinic acid derivative	✓	✓		
3	2.1	191	MS^2^ [191]: 173 (34), 127 (8), 111 (100)	Isocitric acid *	✓	✓	✓	✓
4	2.6	191	MS^2^ [191]: 173 (31), 111 (100)	Citric acid *	✓	✓	✓	✓
5	3.7	315	MS^2^ [315]: 153 (100), 135 (11) MS^3^ [315→153]: 123 (13), 109 (100)	Dihydroxybenzoic acid-*O*-hexoside	✓	✓	✓	✓
6	3.9	395	MS^2^ [395]: 197 (100), 179 (13), 135 (7) MS^3^ [395→197]: 179 (100), 153 (9), 135 (7) MS4 [395→197→179]: 135 (100)	Danshensu (dimer)	✓	✓	✓	✓
7	4.4	353	MS^2^ [353]: 191 (18), 179 (43), 173 (100), 135 (10)	Caffeolylquinic acid	✓			
8	9.0	353	MS^2^ [353]: 191 (18), 179 (37), 173 (100), 135 (8)	4-*O*-caffeoylquinic acid *	✓	✓		
9	9.3	367	MS^2^ [367]: 193 (100), 173 (27), 149 (5), 134 (12) MS^3^ [367→193]: 149 (55), 134 (100)	3-Feruloylquinic acid	✓	✓	✓	
10	9.7	305	MS^2^ [305]: 225 (100) MS^3^ [305→225]: 147 (95), 135 (100)	Unknown			✓	✓
11	10.6	387	MS^2^ [387]: 207 (100), 163 (33), 119 (8), 113 (15) MS^3^ [387→207]: 163 (100), 145 (3)	Medioresinol	✓	✓	✓	✓
12	11.3	489	MS^2^ [489]: 295 (33), 235 (61), 193 (100), 175 (20) MS^3^ [489→193]: 178 (63), 149 (100), 134 (57)	Ferulic acid derivative	✓	✓		
13	11.3	179	MS^2^ [179]: 135 (100)	Caffeic acid *	✓	✓	✓	✓
14	12.4	609	MS^2^ [609]: 447 (100) MS^3^ [609→447]: 285 (100)	Flavonoid-*O*-dihexoside	✓	✓		
15	12.9	593	MS^2^ [593]: 503 (28), 473 (100), 383 (16), 353 (41) MS^3^ [593→473]: 383 (17), 353 (100)	Vicenin-2 (apigenin-6,8-di-*C*-glucoside) *	✓	✓	✓	✓
16	13.8	367	MS^2^ [367]: 173 (100), 193 (6) MS^3^ [367→173]: 111 (100)	4-Feruloylquinic acid	✓	✓		
17	14.1	473	MS^2^ [473]: 295 (20), 235 (13), 193 (100), 175 (61) MS^3^ [473→193]: 149 (13), 134 (100)	Ferulic acid derivative	✓	✓		
18	14.8	449	MS^2^ [449]: 287 (100) MS^3^ [449→287]: 151 (100), 135 (11), 125 (3), 107 (5)	Eriodictyol-*O*-hexoside	✓			
19	15.0	367	MS^2^ [367]: 191 (100), 173 (13)	5-Feruloylquinic acid		✓		
20	15.1	563	MS^2^ [563]: 545 (45), 503 (75), 473 (100), 443 (25), 383 (45), 353 (54)	Apigenin-6-*C*- pentoside-8-*C*- hexoside	✓	✓		
21	15.3	593	MS^2^ [593]: 503 (20), 473 (40), 431 (100), 353 (36), 311 (40) MS^3^ [593→431]: 341 (9), 311 (100)	Vitexin-hexoside (apigenin di-hexoside)	✓	✓	✓	✓
22	16.0	739	MS^2^ [739]: 431 (100), 311 (28) MS^3^ [739→431]: 341 (19), 311 (100)	Vitexin-rutinoside (apigenin-hexoside-rutinoside)			✓	
23	17.7	477	MS^2^ [477]: 301 (100)	Unknown	✓	✓	✓	✓
24	17.9	563	MS^2^ [563]: 545 (15), 503 (8), 473 (47), 443 (100), 383 (22), 353 (30)	Apigenin-6-*C*- hexoside-8-*C*-pentoside	✓		✓	✓
25	18.0	595	MS^2^ [595]: 287 (100) MS^3^ [595→287]: 151 (100), 135 (25), 125 (8), 107 (12)	Eriodictyol-*O*-rutinoside	✓	✓	✓	✓
26	18.2	377	MS^2^ [377]: 331 (100), 179 (16) MS^3^ [377→331]: 179 (100), 161 (18), 143 (33), 131 (22), 119 (9), 113 (21), 101 (12)	Hexoside derivative	✓	✓	✓	✓
27	18.6	447	MS^2^ [447]: 285 (100) MS^3^ [447→285]: 243 (45), 241 (27), 217 (100), 151 (19)	Luteolin-*O*-hexoside	✓	✓		
28	19.2	431	MS^2^ [431]: 341 (6), 311 (100), 283 (6)	Vitexin (8-*C*-glucosyl apigenin)			✓	✓
29	19.8	303	MS^2^ [303]: 285 (100), 177 (12), 125 (12)	Taxifolin	✓		✓	✓
30	20.4	593	MS^2^ [593]: 285 (100)	Flavonoid-rutinoside	✓	✓	✓	
31	21.2	463	MS^2^ [463]: 301 (100) MS^3^ [463→301]: 255 (16), 229 (12), 179 (64), 151 (100)	Quercetin-*O*-hexoside	✓	✓		
32	21.7	461	MS^2^ [461]: 285 (100) MS^3^ [461→285]: 285 (100), 243 (6), 241 (14)	Luteolin-*O*-glucuronide	✓	✓	✓	✓
33	22.1	521	MS^2^ [521]: 359 (100) MS^3^ [521→359]: 223 (4), 197 (100), 179 (22), 161 (20), 135 (32)	Rosmarinic acid-*O*-hexoside	✓	✓		✓
34	22.2	579	MS^2^ [579]: 271 (100) MS^3^ [579→271]: 151 (100), 125 (12)	Naringenin-*O*-hexoside	✓	✓		
35	22.5	431	MS^2^ [431]: 269 (100) MS^3^ [431→269]: 225 (100), 183 (88), 151 (57)	Apigenin-*O*-hexoside	✓	✓		
36	22.7	579	MS^2^ [579]: 417 (100) MS^3^ [579→417]: 402 (17), 387 (4), 181 (100), 166 (34), 151 (12)	Syringaresinol-*O*-hexoside			✓	✓
37	23.0	623	MS^2^ [623]: 447 (100) MS^3^ [623→447]: 315 (92), 271 (33), 163 (44), 151 (100) MS4 [623→447→271]: 151 (100)	Naringenin-di-*O*-glucuronide	✓	✓		
38	23.5	461	MS^2^ [461]: 299 (100) MS^3^ [461→299]: 284 (100)	Methylated flavonoid-*O*-hexoside	✓	✓		
39	24.0	577	MS^2^ [577]: 269 (100)	Unknown	✓			✓
40	24.3	609	MS^2^ [609]: 301 (100) MS^3^ [609→301]: 286 (100), 242 (21)	Hesperidin (hesperetin 7-*O*-rutinoside) *	✓	✓	✓	✓
41	25.6	607	MS^2^ [607]: 299 (100), 284 (42)	Methylated flavonoid-*O*-rutinoside			✓	✓
42	26.1	359	MS^2^ [359]: 223 (13), 197 (27), 179 (41), 161 (100), 133 (15)	Rosmarinic acid	✓	✓	✓	✓
43	26.7	555	MS^2^ [555]: 493 (100), 359 (54) MS^3^ [555→493]: 359 (100) MS4 [555→493→359]: 197 (21), 179 (14), 161 (100)	Salvianolic acid K	✓	✓		
44	29.2	717	MS^2^ [717]: 555 (18), 519 (100), 357 (62) MS^3^ [→]: MS4 [→→]:	Salvianolic acid B/E isomer		✓	✓	✓
45	29.6	537	MS^2^ [537]: 493 (100), 359 (24) MS^3^ [537→493]: 359 (100), 179 (12), 161 (10)	Salvianolic acid I (lithospermic acid A)	✓			
46	30.7	717	MS^2^ [717]: 519 (100) MS^3^ [717→519]: 339 (24), 321 (100)	Salvianolic acid B/E isomer			✓	✓
47	31.1	505	MS^2^ [505]: 193 (100) MS^3^ [505→193]: 149 (18), 134 (100)	Ferulic acid derivative	✓		✓	
48	32.4	287	MS^2^ [287]: 151 (100)	Eriodictyol	✓	✓	✓	✓
49	33.5	637	MS^2^ [637]: 591 (100) MS^3^ [637→591]: 283 (100), 268 (12)	Methylated flavonoid-*O*-rutinoside			✓	✓
50	35.5	301	MS^2^ [301]: 179 (100), 151 (84)	Quercetin *			✓	
51	36.1	285	MS^2^ [285]: 285 (100), 243 (7), 241 (46), 151 (11)	Luteolin	✓		✓	
52	36.3	717	MS^2^ [717]: 519 (100) MS^3^ [717→519]: 339 (100)	Salvianolic acid B/E isomer	✓	✓		✓
53	36.9	493	MS^2^ [493]: 359 (100), 313 (10), 161 (23) MS^3^ [493→359]: 223 (12), 197 (23), 179 (26), 161 (100)	Salvianolic acid A	✓	✓		✓
54	38.1	329	MS^2^ [329]: 314 (100) MS^3^ [329→314]: 299 (100)	Dimethylated flavonoid			✓	✓
55	38.2	551	MS^2^ [551]: 519 (79), 359 (100) MS^3^ [551→359]: 223 (27), 197 (35), 179 (13), 161 (100)	Monomethyl lithospermate	✓			
56	39.1	327	MS^2^ [327]: 309 (27), 291 (43), 229 (100), 211 (66)	Oxo-dihydroxy-octadecenoic acid	✓	✓	✓	✓
57	39.2	271	MS^2^ [271]: 151 (100)	Naringenin	✓		✓	
58	40.0	269	MS^2^ [269]: 269 (100), 225 (36), 197 (28), 151 (79):	Apigenin *	✓		✓	
59	40.6	329	MS^2^ [329]: 311 (20), 229 (100), 211 (69), 209 (10), 171 (27)	Trihydroxy-octadecenoic acid	✓	✓	✓	✓

* Compared with standard compound.

**Table 3:** Relative peak areas (%) and heat map for *Thymus sipylus* and *Thymbra capitata*, obtained by HPLC-ESI-MS analysis. Hex = hexoside; Pen = pentoside; Rut = rutinoside.

		*T. sipylus*		*T. capitata*	
Peak	Compound	MeOH	Infusion	Peak	Compound
1	Disaccharide	3.43	0.00	0.66	0.00
2	Quinic acid derivative	2.00	8.72	0.00	0.00
3	Isocitric acid	0.09	0.05	0.05	0.05
4	Citric acid	0.01	0.02	0.01	0.14
5	Dihydroxybenzoic acid-O-Hex	0.25	1.04	0.05	0.23
6	Danshensu	0.42	1.47	0.51	1.26
7	Caffeolylquinic acid	0.02	0.00	0.00	0.00
8	4-*O*-caffeoylquinic acid	0.87	1.21	0.00	0.00
9	3-Feruloylquinic acid	0.30	2.67	0.01	0.00
10	Unknown	0.00	0.00	12.13	32.12
11	Medioresinol	5.09	6.91	0.56	1.61
12	Ferulic acid derivative	0.12	0.50	0.00	0.00
13	Caffeic acid	0.12	0.50	0.11	0.05
14	Flavonoid-*O*-di-Hex	0.33	1.10	0.00	0.00
15	Vicenin-2	3.38	11.55	10.85	13.63
16	4-Feruloylquinic acid	3.89	4.11	0.00	0.00
17	Ferulic acid derivative	0.14	0.30	0.00	0.00
18	Eriodictyol-O-Hex	0.14	0.00	0.00	0.00
19	5-Feruloylquinic acid	0.00	1.78	0.00	0.00
20	Apigenin-6-C-Pen-8-C-Hex	0.10	0.38	0.00	0.00
21	Vitexin Hex	0.16	0.47	0.30	0.59
22	Vitexin-Rut	0.00	0.00	0.08	0.00
23	Unknown	0.74	2.23	0.10	0.32
24	Apigenin-6-C-Hex-8-C-Pen	0.09	0.00	0.17	0.28
25	Eriodictyol-O-Rut	0.58	0.63	2.53	1.55
26	Hexoside derivative	2.33	2.48	0.01	0.39
27	Luteolin-O-Hex	3.34	2.54	0.00	0.00
28	Vitexin	0.00	0.00	0.73	0.56
29	Taxifolin	0.31	0.00	3.67	0.93
30	Flavonoid-Rut	1.04	0.57	4.27	0.00
31	Quercetin-O-Hex	0.38	0.40	0.00	0.00
32	Luteolin-O-Gluc	6.57	11.15	2.43	5.01
33	Rosmarinic acid-O-Hex	0.22	0.51	0.00	0.58
34	Naringenin-O-Hex	0.24	0.30	0.00	0.00
35	Apigenin-O-Hex	1.08	0.89	0.00	0.00
36	Syringaresinol-*O*-Hex	0.00	0.00	1.04	1.36
37	Naringenin-di-*O*-Gluc	1.36	3.13	0.00	0.00
38	Methylated flavonoid-O-Hex	0.85	0.47	0.00	0.00
39	Unknown	1.01	0.00	0.00	0.28
40	Hesperidin	0.18	0.09	10.19	1.87
41	Methylated flavonoid-O-Rut	0.00	0.00	5.85	0.78
42	Rosmarinic acid	25.56	14.96	20.95	22.80
43	Salvianolic acid K	4.44	9.31	0.00	0.00
44	Salvianolic acid B/E isomer	0.00	0.78	2.84	5.79
45	Salvianolic acid I	12.24	0.00	0.00	0.00
46	Salvianolic acid B/E isomer	0.00	0.00	1.84	3.38
47	Ferulic acid derivative	0.36	0.00	0.09	0.00
48	Eriodictyol	2.40	0.25	2.79	0.58
49	Methylated flavonoid-O-Rut	0.00	0.00	6.07	0.57
50	Quercetin	0.00	0.00	0.30	0.00
51	Luteolin	1.08	0.00	2.01	0.00
52	Salvianolic acid B/E isomer	1.54	1.97	0.00	0.15
53	Salvianolic acid A	0.64	1.55	0.00	0.50
54	Dimethylated flavonoid	0.00	0.00	2.64	0.42
55	Monomethyl lithospermate	6.21	0.00	0.00	0.00
56	Oxo-dihydroxy-octadecenoic acid	1.47	2.06	1.08	1.36
57	Naringenin	1.43	0.00	0.25	0.00
58	Apigenin	0.61	0.00	2.11	0.00
59	Trihydroxy-octadecenoic acid	0.86	0.94	0.69	0.85

**Table 4:** Quantification of the main phytochemicals in the analyzed extract of *Thymus sipylus* and *Thymbra capitata* (mg g^−1^ DE; *n* = 3).

No.	Assigned Identification	*T. sipylus*		*T. capitata*	
		MeOH	Inf	MeOH	Inf
*Phenolic acids*					
8 + 9	CQA + FQA	2.4 ± 0.2 ^b^	5.5 ± 0.4 ^a^	---	---
12 + 13	Ferulic + caffeic acids	1.04 ± 0.07 ^a^	0.61 ± 0.04 ^b^	---	---
16	FQA	1.8 ± 0.1 ^a^	1.00 ± 0.07 ^b^	---	---
19	FQA	---	0.49 ± 0.03	---	---
42	Rosmarinic acid	19 ± 1 ^a^	12.3 ± 0.8 ^b^	9.3 ± 0.6 ^c^	6.1 ± 0.4 ^d^
43	Salvianolic acid K	1.07 ± 0.06 ^b^	1.8 ± 0.1 ^a^	---	---
44	Salvianolic acid B/E	---	0.29 ± 0.02 ^b^	0.31 ± 0.02 ^b^	0.46 ± 0.03 ^a^
45	Salvianolic acid I	5.4 ± 0.4	---	---	---
46	Salvianolic acid B/E	---	---	0.25 ± 0.02 ^b^	0.43 ± 0.03 ^a^
53	Salvianolic acid A	0.34 ± 0.02 ^a^	0.42 ± 0.03 ^a^	---	0.35 ± 0.02 ^a^
55	Monomethyl Lith	1.34 ± 0.08	---	---	---
Total		32 ± 1 ^a^	22.4 ± 0.9 ^b^	9.9 ± 0.6 ^c^	7.3 ± 0.4 ^d^
*Flavonoids*					
15	Vicenin-2	2.5 ± 0.1 ^c^	6.8 ± 0.4 ^b^	7.0 ± 0.4 ^b^	9.0 ± 0.5 ^a^
20 + 21	Apigenin glycosides	---	0.29 ± 0.02 ^b^	0.27 ± 0.02 ^b^	0.58 ± 0.04 ^a^
25	Eriodictyol-*O*-rutinoside	---	---	0.43 ± 0.03 ^a^	0.46 ± 0.03 ^a^
27	Luteolin-*O*-hexoside	5.5 ± 0.4 ^a^	2.2 ± 0.1 ^b^	---	---
28	Vitexin	---	---	0.33 ± 0.02 ^a^	0.36 ± 0.02 ^a^
29	Taxifolin	0.50 ± 0.04 ^b^	---	1.18 ± 0.07 ^a^	---
31	Quercetin-*O*-hexoside	---	0.24 ± 0.02	---	---
32	Luteolin-*O*-glucuronide	13.1 ± 0.8 ^a^	7.4 ± 0.5 ^b^	1.6 ± 0.1 ^d^	2.1 ± 0.1 ^c^
37	Naringenin-di-*O*-Gluc	1.14 ± 0.08 ^a^	0.19 ± 0.01 ^b^	---	---
40	Hesperidin	---	---	0.51 ± 0.03	---
48	Eriodictyol	0.41 ± 0.03 ^a^	---	0.20 ± 0.01 ^b^	0.44 ± 0.03 ^a^
57	Naringenin	0.35 ± 0.02	---	---	---
58	Apigenin	0.38 ± 0.03 ^b^	---	2.2 ± 0.1 ^a^	---
Total		24 ± 1 ^a^	17.1 ± 0.7 ^b^	13.7 ± 0.4 ^c^	12.9 ± 0.5 ^c^
TIPC		56 ± 2 ^a^	40 ± 1 ^b^	23.6 ± 0.7 ^c^	20.2 ± 0.6 ^d^

CQA = caffeoylquinic acid; FQA = feruloylquinic acid; Lith = lithospermate; Gluc = glucuronide. Different letters indicate significant differences in the tested extracts (*p* < 0.05). TIPC = total individual phenolic compounds (sum of all the compounds quantified by HPLC).

**Table 5:** Antioxidant properties of the tested extracts.

Extracts	CUPRAC (mg TE/g)	FRAP (mg TE/g)	Metal Chelating (mg EDTAE/g)	Phosphomolybdenum (mmol TE/g)
*T. capitata*-Methanol	802.22 ± 34.70 ^a^	270.16 ± 6.75 ^b^	16.61 ± 0.96 ^c^	3.61 ± 0.27 ^a^
*T. capitata*-Infusion	622.65 ± 15.73 ^c^	285.42 ± 5.70 ^a^	28.04 ± 2.80 ^b^	2.57 ± 0.08 ^b^
*T. sipylus*-Methanol	657.70 ± 3.05 ^b^	249.33 ± 8.21 ^c^	14.97 ± 3.00 ^d^	2.52 ± 0.23 ^b^
*T. sipylus*-Infusion	625.20 ± 16.29 ^c^	278.37 ± 5.64 ^a^	36.72 ± 0.73 ^a^	2.39 ± 0.11 ^c^

Values are reported as mean ± S.D of three parallel measurements. TE: trolox equivalent; EDTAE: EDTA equivalent. Different letters indicate significant differences in the tested extracts (*p* < 0.05).

**Table 6:** Enzyme inhibitory properties of tested extracts.

Extracts	AChE (mg GALAE/g)	BChE (mg GALAE/g)	Tyrosinase (mg KAE/g)	Amylase (mmol ACAE/g)	Glucosidase (mmol ACAE/g)
*T. capitata*-Methanol	3.86 ± 0.35 ^a^	4.36 ± 0.37 ^a^	89.66 ± 0.66 ^a^	0.84 ± 0.03 ^a^	1.78 ± 0.03 ^a^
*T. capitata*-Infusion	0.73 ± 0.02 ^c^	na	19.11 ± 3.69 ^b^	0.11 ± 0.01 ^c^	1.67 ± 0.03 ^b^
*T. sipylus*-Methanol	3.49 ± 0.14 ^b^	3.79 ± 0.12 ^b^	83.18 ± 2.57 ^a^	0.61 ± 0.07 ^b^	1.73 ± 0.04 ^a^
*T. sipylus*-Infusion	0.35 ± 0.05 ^d^	na	18.74 ± 2.24 ^b^	0.11 ± 0.01 ^c^	1.45 ± 0.03 ^c^

Values are reported as mean ± S.D of three parallel measurements. GALAE: galantamine equivalent; KAE: kojic acid equivalent; ACAE: acarbose equivalent. na: not active. Different letters indicate significant differences in the tested extracts (*p* < 0.05).

## Data Availability

Not applicable.
